# Supplementary material for: Dual targeting of BRAFV600E and ferroptosis results in synergistic anticancer activity via iron overload and enhanced oxidative stress
Source: J Exp Clin Cancer Res. 2026 Jan 7;45:34. doi: 10.1186/s13046-025-03624-z (PMC12871017; doi:10.1186/s13046-025-03624-z)
Supplement: Supplementary file 1 — Supplementary Material 1. Fig S1. Erastin treatment of ATC cell lines. Fig S2. Effect of dabrafenib and RSL3 combination on the MEK pathway. Fig S3. Ferroptosis inhibition reverses the growth-suppressive effects of C18 alone and C18 combined with dabrafenib in BRAFV600E-mutant ATC cells. Ferrostatin-1 (2 µM) and liproxstatin-1 (2 µM) are ferroptosis inhibitors that block lipid peroxidation. ATC cells were treated with C18 (25 nM), dabrafenib (5 µM), ferrostatin-1 (2 µM), or liproxstatin-1 (2 µM) for 48 h. Proliferation was measured using the CyQUANT Cell Proliferation Assay. (A) The reduction in cell proliferation caused by C18 treatment is rescued by co-treatment with ferrostatin-1 or liproxstatin-1. (B) The antiproliferative effect of combined C18 and dabrafenib is similarly reversed by ferrostatin-1 or liproxstatin-1. Statistical analysis was performed using one-way ANOVA in GraphPad Prism. Significance: *P < 0.05; **P < 0.01; ***P < 0.001; ns = nonsignificant. Fig S4. Representative bioluminescence images of mice depicting tumor luciferase intensity at 14 days post-treatment. Table S1. Dabrafenib (D) and RSL3 (R) combination shows synergistic activity on BRAFV600E-mutant thyroid cancer cells. Table S2. The list of primary and secondary antibodies used in the study. [file 13046_2025_3624_MOESM1_ESM.pdf]

Supplementary Materials

Supplementary Figure S1

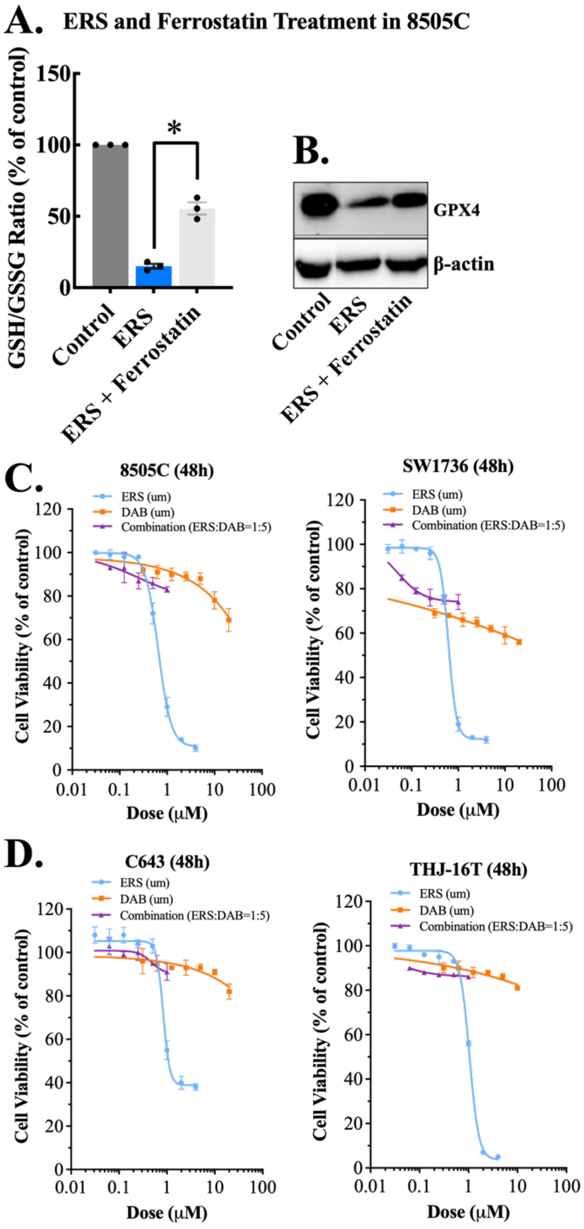

Supplementary Fig S1. Erastin treatment in ATC cells. (A) The GSH/GSSG ratio and (B) expression level of GPX4 were reduced with erastin (2 μM) in a *BRAF*^{V600E}-mutant 8505C ATC cell line for 48h, which was partially recovered by ferrostatin-1 (a ferroptosis inhibitor, 2 μM), confirming induction of ferroptosis in the ATC cells. (C) The cell viability curves of combinatorial treatment in *BRAF*^{V600E}-mutant (C) and *BRAF*^{WT} (D) ATC cell lines were plotted according to the doses of RSL3 used in the treatment. *p < 0.05, ns = nonsignificant. All data were presented as mean ± SEM (n = 3).

Supplementary Figure S2

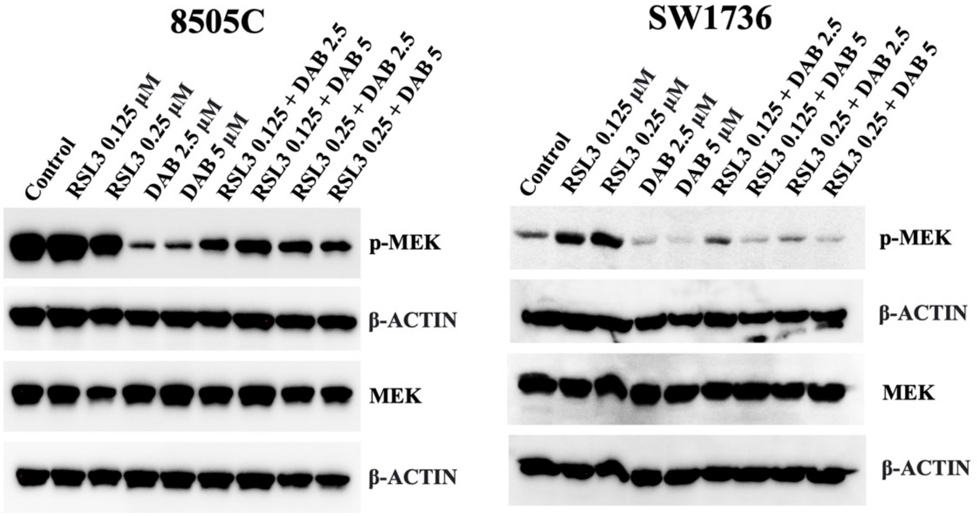

Supplementary Fig S2. Effect of dabrafenib and RSL3 combination on MEK pathway.

Supplementary Figure S3

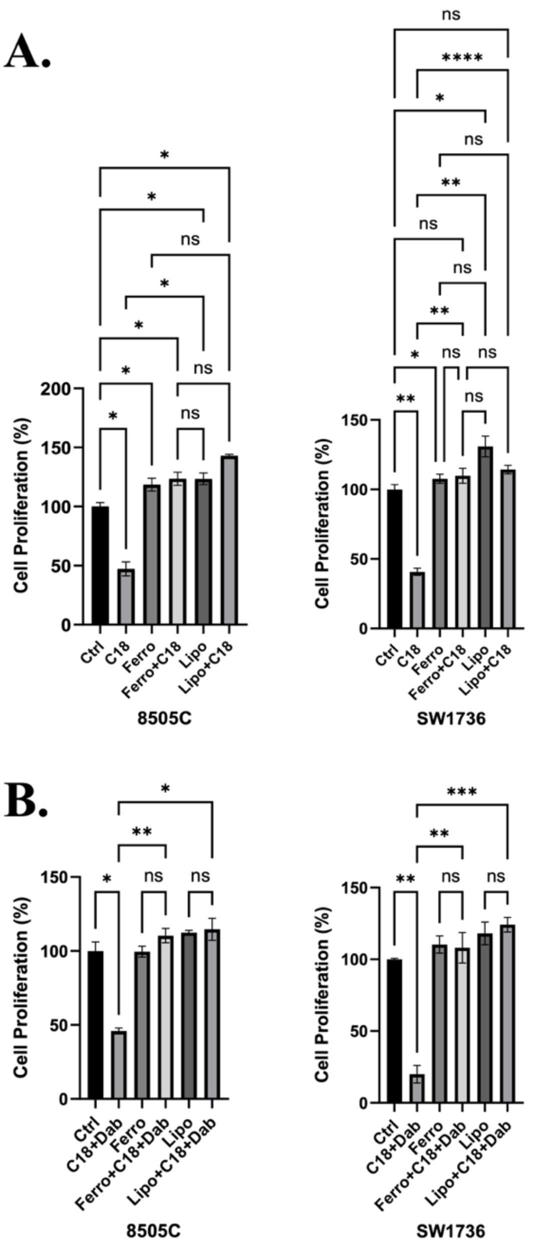

Supplementary Fig S3. Ferroptosis inhibition reverses the growth-suppressive effects of C18 alone and C18 combined with dabrafenib in BRAF-mutant ATC cells. Ferrostatin-1 (2 μM) and liproxstatin-1 (2 μM) are ferroptosis inhibitors that block lipid peroxidation. ATC cells were treated with C18 (25 nM), dabrafenib (5 μM), ferrostatin-1 (2 μM), or liproxstatin-1 (2 μM) for 48 h. Proliferation was measured using the CyQUANT Cell Proliferation Assay. (A) The reduction in cell proliferation caused by C18 treatment is rescued by co-treatment with ferrostatin-1 or liproxstatin-1. (B) The antiproliferative effect of combined C18 and dabrafenib is similarly reversed by ferrostatin-1 or liproxstatin-1. Statistical analysis was performed using one-way ANOVA in GraphPad Prism. Significance: *P < 0.05; **P < 0.01; ***P < 0.001; ns = nonsignificant.

Supplementary Figure S4

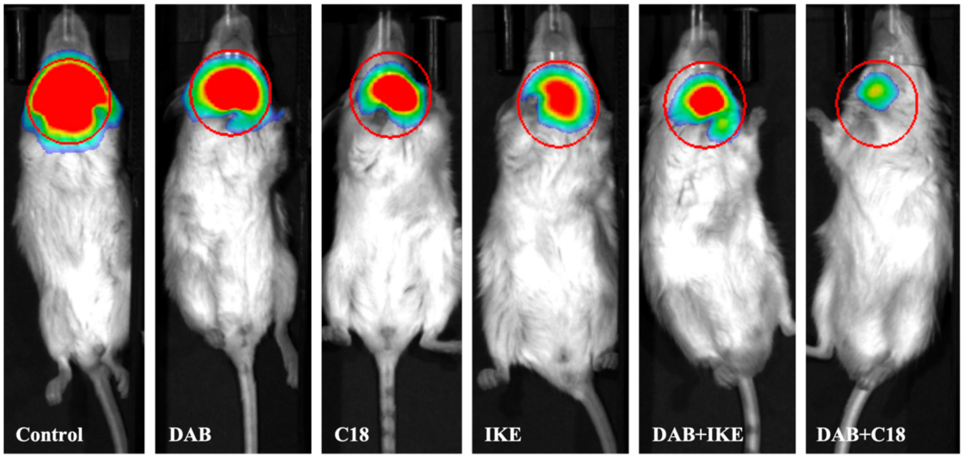

Supplementary Fig S4. Representative bioluminescence images of mice depicting tumor luciferase intensity at 14 days post-treatment. DAB = dabrafenib, IKE = imidazole ketone erastin.

Supplementary Table S1

Supplemental Table S1. Dabrafenib (D) and RSL3 (R) combination shows synergistic activity on *BRAF*^{V600E}-mutant thyroid cancer cells.

Cell lines	D10 + R0.5 µM	D10 + R0.25 µM	D10 + R0.125 µM	D5 + R0.5 µM	D5 + R0.25 µM	D5 + R0.125 µM	D2.5 + R0.25 µM	D2.5 + R0.125 µM
8505C	1.0056	1.0901	**0.4637**	1.5798	**0.5336**	**0.3518**	**0.2694**	**0.2018**
SW1736	3.0365	2.3083	1.8999	**0.3299**	**0.4100**	**0.3700**	**0.4700**	**0.3700**
C643	4.3423	1.8103	1.0835	3.0745	2.1360	1.4322	1.0653	1.2184
THJ-16T	1.6498	1.1602	1.0063	2.1657	4.5079	5.7678	15.516	9.8255

The combination index (CI) was calculated using the Chou–Talalay method with the combination dabrafenib and RSL3 treatment at 48 h. Dabrafenib (5 µM, 2.5 µM) in combination with RSL3 (0.25µM, 0.125µM) showed synergistic effects in the *BRAF*^{V600E}-mutant cell lines (8505C and SW1736). In the *BRAF*^{WT} cell lines (C643 and THJ-16T), however, there were mostly antagonistic effects in the combination treatment. The CI was interpreted as follows: < 1, synergist (bold); 1, additive; > 1, antagonist.

Supplementary Table S2

**Supplemental Table S2. The list of primary and secondary antibodies used in the immunoblotting study.**		
**Antibody Name**	**Catalog Number**	**Sources**
Phospho-ERK	9101	Cell Signaling Technology
Total ERK	9102	Cell Signaling Technology
Phospho-MEK1/2	2338S	Cell Signaling Technology
MEK1/2 (L38C12)	4694S	Cell Signaling Technology
GPX4	52455S	Cell Signaling Technology
Ferroportin (FPN1)	PA5-22993	Invitrogen
Transferrin receptor 1 (CD71, TFRC)	13113	Cell Signaling Technology
DMT1	ab55735	Abcam
FTH1	4393	Cell Signaling Technology
Keap1	ab119403	Abcam
Nrf2	12721S	Cell Signaling Technology
HO-1	70081	Cell Signaling Technology
β-Actin (13E5) Rabbit mAb	4970	Cell Signaling Technology
Anti-rabbit IgG, HRP linked	7074P2	Cell Signaling Technology
Anti-mouse IgG, HRP linked	7076P2	Cell Signaling Technology
